# Clinicopathological characteristics of primary peritoneal epithelioid mesothelioma of clear cell type: A case report

**DOI:** 10.1097/MD.0000000000025264

**Published:** 2021-03-26

**Authors:** Xue-Mei Du, Ya-Ping Wei, Ying Gao, Zhao Li, Jian-Mei Zhang, Hong Chang, Yan Li

**Affiliations:** a Department of Pathology; b Department of Radiology; c Department of Peritoneal Cancer Surgery, Beijing Shijitan Hospital, Capital Medical University, Beijing, China.

**Keywords:** clear cell type, immunohistochemistry, primary peritoneal epithelioid mesothelioma, whole-exome sequencing

## Abstract

**Rationale:**

Primary peritoneal epithelioid mesothelioma of clear cell type is an extremely rare entity composed of clear cytoplasm. It is challenging to diagnose because of the morphological resemblance to clear cell tumor.

**Patient's concerns:**

A 69-year-old male patient had swollen lymph nodes in the right inguinal region for 7 months and was constipated for 1 month.

**Diagnosis:**

The patient was diagnosed as peritoneal epithelioid mesothelioma of clear cell type based on computed tomography scan, pathology, immunohistochemistry, special staining and whole-exome sequencing. This patient harbored *VHL* gene alteration in exon 1 and homologous recombination defect (with a score of 45). This finding indicated that this patient might be sensitive to platinum-based therapy and Poly ADP-ribose Polymerase (PARP) inhibitor. This patient carried no microsatellite instability, a low level of tumor mutation burden, and a high extent of intratumoral heterogeneity. Eighteen neoantigens were detected.

**Interventions:**

The patient received surgery-based multidisciplinary treatment by integrating cytoreductive surgery (CRS) with hyperthermic intraperitoneal chemotherapy (HIPEC). HIPEC was administered with docetaxel 120 mg plus cisplatin 120 mg, at 43°C, for 60 minutes. After operation, the patient received intravenous (IV) chemotherapy with docetaxel 60 mg, pemetrexed 750 mg and cisplatin 100 mg, and then intraperitoneal (IP) chemotherapy with docetaxel 40 mg. The patient received interventional therapy of hepatic artery embolization for 5 times.

**Outcomes:**

Regular follow-up was performed until Oct 14, 2020. The patient died 31.6 months later owing to incomplete intestinal obstruction.

**Lessons:**

Primary peritoneal epithelioid mesothelioma of clear cell type needs to be differentiated from a variety of clear cell tumors. This disease is characterized by specific genetic alteration. Whole-exome sequencing contributes to guide individualized therapy. CRS-HIPEC helps achieve long-term overall survival.

## 1 Introduction

Malignant mesothelioma (MM) involves serosal surfaces, which originates from mesothelium comprising the pleura, peritoneum, pericardium and tunica vaginalis testis. Diffuse malignant peritoneal mesothelioma (DMPM) accounts for 7% to 30% of all cases.^[1]^ Generally, DMPM is a rare and aggressive primary peritoneal malignancy, characterized by widespread multiple metastatic tumorous nodules originating from the peritoneum. The DMPM exhibits 3 major histologic subtypes, divided into epithelioid, sarcomatoid, or mixed (biphasic) categories in the updated 2015 World Health Organization classification.^[2]^ The epithelioid type of mesothelioma frequently contains papillary or tubular components; however, clear cell variant is an extremely rare entity, which has only been described in a few case reports.^[3,4]^ Diagnostic difficulties may be encountered because unusual clear cell morphological variants of mesothelioma can be confused with a variety of other clear cell tumors. We report on mesothelioma presenting as clear cell morphology, in attempt to explore differential diagnosis, molecular pathology and prognosis.

## 2 Case presentation

A 69-year-old male patient was diagnosed with primary peritoneal epithelioid mesothelioma of clear cell type. He had swollen lymph nodes in the right inguinal region for 7 months and dyssynergic defecation for 1 month, who presented to our hospital in October 2018. He had a history of asbestos exposure and a family history of cancer.

### 2.1 CT enhancement and 3D reconstruction imaging

**Figure 1:**
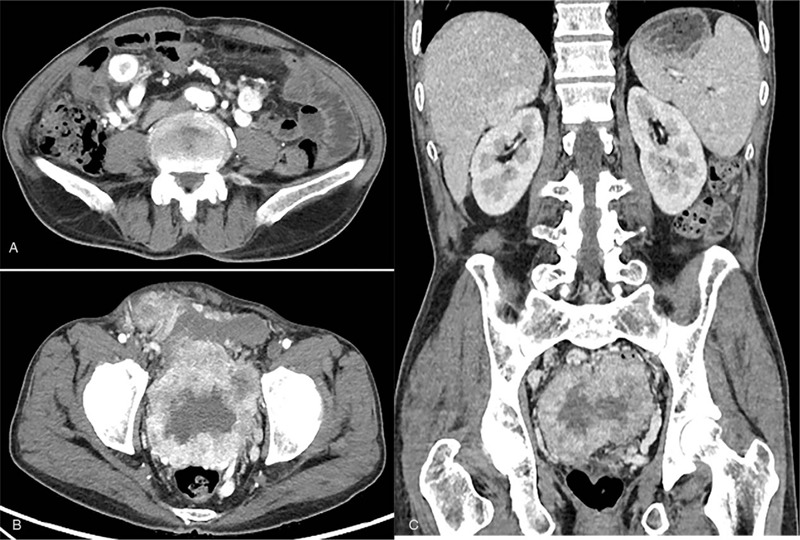
CT enhancement and 3D reconstruction imaging. (A) Multiple nodules in the abdominal cavity. (B) A huge tumor in the pelvic cavity. (C) Inhomogeneous strengthening.

The shape of the left kidney was normal. By contrast, multiple cystic low-density shadows were present in the right renal parenchyma, without obvious enhancement on enhanced scanning. Multiple nodules were identified in the abdominal cavity (Fig. 1A). A huge tumor was identified in the pelvic cavity (Fig. 1B) with obvious inhomogeneous enhancement (Fig. 1C).

### 2.2 Intraoperative findings

**Figure 2:**
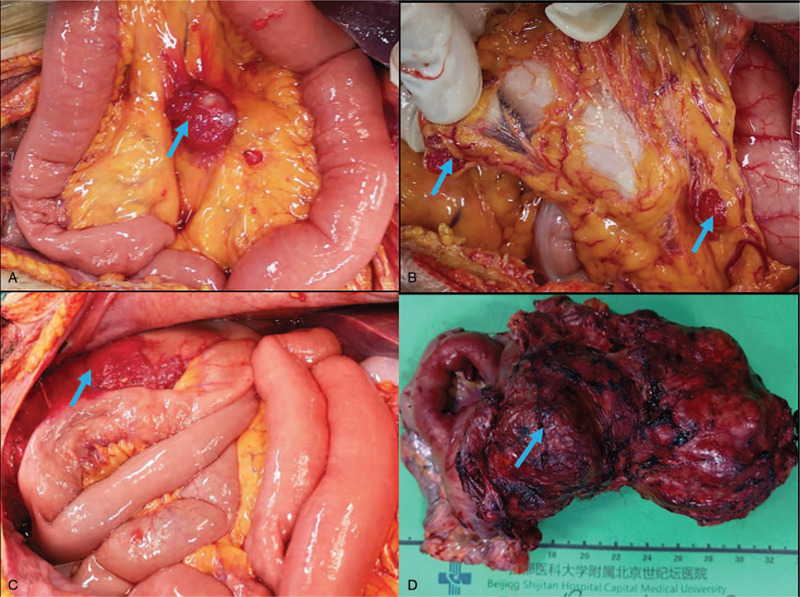
Intraoperative findings. (A) Tumor nodules in mesentery. (B)Tumor nodules in the greater omentum. (C) Huge tumors located in the pelvic cavity. (D) Pelvic tumors.

Tumor nodules (with the maximum diameter of 3.5 cm) were scattered on the surface of small intestinal mesentery (Fig. 2A). Tumor nodules were universally distributed on the surface of ligamentum teres and greater omentum, with the maximum diameter of 2 cm (Fig. 2B). Patchy tumor nodules were scattered in bilateral subphrenic peritoneum, with the largest size of 6 × 6 × 0.8 cm. Additionally, tumor nodules were present in the para-colonic sulcus on both sides, with maximum diameter of 3 cm on the left amd 2 cm on the right. The peritoneum of the left lower abdominal wall was adhered to a part of sigmoid colon, harboring scattered small tumor nodules with invasion to rectum and mesorectum. The small intestine, mesentery, ileocecal and pelvic masses (Fig. 2C, D) formed a dense adhesion at 400 cm from flexor ligament. The PCI (peritoneal cancer index) score was 24. The CCR (completeness of cytoreduction) was minimal residual disease.

### 2.3 Gross examination

**Figure 3:**
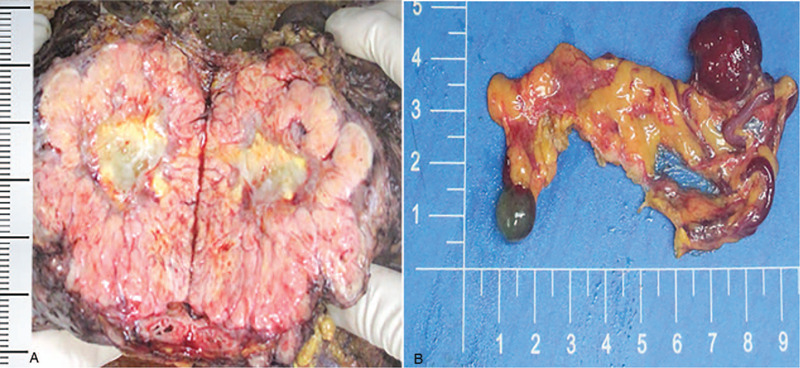
Gross examination. (A) A huge (18 × 15 × 11 cm) solid tumor was located in pelvic cavity, with grayish yellow section. (B) Two soft masses (2 × 2 × 0.3 cm and 1 × 0.8 × 0.5 cm, respectively) located in the omentum, with grayish red section.

A huge (18 × 15 × 11 cm) tumor was located in pelvic cavity (Fig. 3A). The section was grayish yellow. The texture was solid and medium. Two masses were located in the omentum (2 × 2 × 0.3 cm, 1 × 0.8 × 0.5 cm, respectively) (Fig. 3B). The section was grayish red and soft.

### 2.4 Histopathological analysis

Removed surgical specimens were fixed in 10% phosphate-buffered, neutral formaldehyde solution at room temperature for 24 hours and dehydrated in an ascending series of ethanol. Samples were routinely embedded in paraffin, washed with xylene, rehydrated in a descending series of alcohol, washed with distilled water, and then stained with hematoxylin and eosin for 30 minutes at room temperature. Sections (4-μm thick) were observed under a light microscope with magnification of ×40, ×100, ×200, and ×400, respectively.

**Figure 4:**
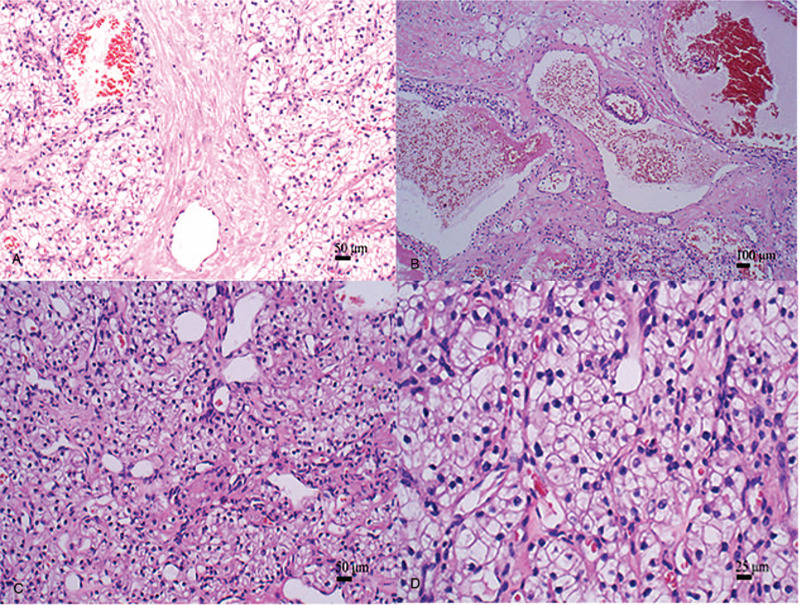
Histopathological characteristics. (A) Most tumor cells were arranged in sheet-like structures (magnification, ×100). (B) A minority of tumor cells were arranged in tubular structures (magnification, ×100). (C) Tumor cells had clear cytoplasm (magnification, ×200). (D) The tumor was composed of large round to polygonal cells. Tumor cells displayed abundant clear cytoplasm with evident cytoplasmic membranes and eccentric small round nuclei (magnification, ×400).

Microscopically, most tumor cells were arranged in sheet-like structures without papillary patterns (Fig. 4A). Some tumor cells were arranged in tubular structures (Fig. 4B). Tumor cells had clear cytoplasm (Fig. 4C). The tumor was composed of large round to polygonal cells. Tumor cells displayed abundant clear cytoplasm with evident cytoplasmic membrane and eccentric small round nuclei (Fig. 4D). Mitotic figures were rare.

### 2.5 Immunohistochemical markers

Removed surgical specimens were fixed in 10% phosphate-buffered, neutral formaldehyde solution at room temperature for 24 hours. Tissue sections (4-μm thick) were deparaffinized, rehydrated and antigen retrieved with working solution of EnVision FLEX Target Retrieval solution High Ph (50 × ) according to the manufacturer's protocol [EnVision FLEX+, Mouse, high Ph (Link) HRP; # K8002; Dako; Agilent Technologies, Inc., Santa Clara, CA, USA] in PT Link (#PT100; Dako; Agilent Technologies, Inc.) at 95°C for 20 minutes, and washed in distilled water.^[5]^

Endogenous peroxidase was blocked by DAKO EnVision FLEX peroxidase-blocking reagent for 10 minutes, and washed for 3 times in the PBS Wash buffer (Origene Technologies, Inc., Wuxi, China). The slides were incubated for 20 to 30 minutes at room temperature in humidity chamber with appropriate dilutions of primary antibodies (primary antibodies were detailed in Table 1) along with positive and negative controls. Immunohistochemistry was performed by automatic immunohistochemical instrument (intelliPATH FLX, Beijing Zhongshan Jinqiao Biotechnology Co., Ltd). The sections (4-μm thick) were incubated with secondary antibody (MA-2000, Origene Technologies, Inc., Wuxi, China) for coupling reaction for 20 to 30 minutes at room temperature. The substrate (EnVision FLEX DAB+ Chromogen) was used to produce crisp brown color at the site of target antigen. Then, hematoxylin (1–2 dips) was used as a counter stain. Sections were observed under a light microscope with magnification of ×40, ×100, ×200, and ×400, respectively.

**Table 1:** Primary antibodies used for immunohistochemistry.

Target	Supplier	Catalog number	Dilution	Staining
CK	Origene Technologies, Inc.	ZM-0069	1:80	+
EMA	Gene Tech Co., Ltd.	GM061329	1:200	+
CK7	Origene Technologies, Inc.	ZA-0573	1:200	+
CAM5.2	Origene Technologies, Inc.	ZM-0316	Ready to use	–
CK20	Origene Technologies, Inc.	ZA-0574	1:60	–
CK8/18	Origene Technologies, Inc.	ZM-0315	1:80	–
CK5/6	Origene Technologies, Inc.	ZM-0313	Ready to use	–
Vimentin	Gene Tech Co., Ltd.	GM072529	1:120	+
Calretinin	Origene Technologies, Inc.	TA353630	1:60	Focal +
WT-1	Gene Tech Co., Ltd.	GM356102	Ready to use	+
D2-40	Gene Tech Co., Ltd.	GM361929	1:60	+
MC	Gene Tech Co., Ltd.	ZM-0386	Ready to use	–
CD10	Origene Technologies, Inc.	ZA-0526	Ready to use	–
RCC	Origene Technologies, Inc.	ZM-0159	Ready to use	–
CD15	Origene Technologies, Inc.	ZM-0037	Ready to use	–
PAX8	Origene Technologies, Inc.	ZM-0468	Ready to use	–
CDX-2	Origene Technologies, Inc.	ZM-0520	Ready to use	–
SATB2	Origene Technologies, Inc.	ZM-0163	Ready to use	–
CDH17	Origene Technologies, Inc.	ZA-0630	Ready to use	–
GATA3	Origene Technologies, Inc.	ZA-0661	Ready to use	–
CEA	Origene Technologies, Inc.	ZA-0662	Ready to use	–
B72.3	Origene Technologies, Inc.	ZM-0024	Ready to use	–
Ber-EP4	Origene Technologies, Inc.	ZM-0099	Ready to use	–
GPC3	Origene Technologies, Inc.	ZM-0146	Ready to use	–
Hepatocyte	Origene Technologies, Inc.	ZM-0131	Ready to use	–
HMB45	Origene Technologies, Inc.	ZM-0187	Ready to use	–
Melan-A	Origene Technologies, Inc.	ZM-0398	Ready to use	–
S100	Origene Technologies, Inc.	Polyclonal	Ready to use	–
a-inhibin	Origene Technologies, Inc.	ZM-0460	Ready to use	–
CD117	Origene Technologies, Inc.	ZA-0523	Ready to use	–
Dog-1	Origene Technologies, Inc.	ZM-0371	Ready to use	–
Erg	Origene Technologies, Inc.	ZA-0545	Ready to use	–
FLI-1	Origene Technologies, Inc.	ZM-0108	Ready to use	–
P63	Origene Technologies, Inc.	ZM-0406	Ready to use	–
SMA	Origene Technologies, Inc.	ZM-0003	Ready to use	–
Desmin	Origene Technologies, Inc.	ZA-0610	1:60	Focally +
Ki67	Origene Technologies, Inc.	UM870033	1:100	Index 20%
P53	Gene Tech Co., Ltd.	GM700101	Ready to use	+
DES	Origene Technologies, Inc.	TA502328	1:60	Focally +
TTF-1	Origene Technologies, Inc.	ZA-0270	Ready to use	–
INI-1	Origene Technologies, Inc.	ZM-0173	Ready to use	+

CK = cytokeratins, DES = desmin, DOG1 = anoctamin-1, EMA = epithelial membrane antigen, MC = mesothelial cells, SMA = smooth muscle actin, WT-1 = Wilms’ tumor 1.

**Figure 5:**
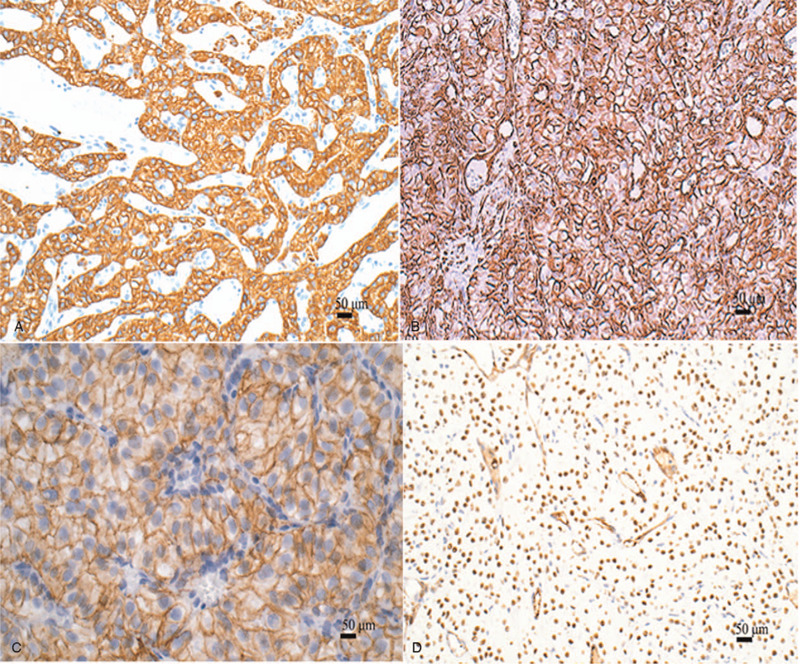
Immunohistochemical staining. (A) Cytoplasmic staining for CK (magnification, ×200). (B) Cytoplasmic staining for Vimentin (magnification, ×200). (C) Cell membrane staining for D2–40 (magnification, ×200). (D) Nuclear staining for WT-1 (magnification, ×200).

Immunohistochemical staining is depicted in Table 1. Tumor cells exhibited diffuse strong staining for cytokeratins (CKs) (Fig. 5A), Vimentin (Fig. 5B), D2–40 (Fig. 5C), Wilms’ tumor 1 (WT-1) (Fig. 5D) and INI-1. Tumor cells were focally positive for calretinin. The Ki67 index was 20%. There was no reactivity to CAM5.2, CK20, CK8/18, MC, CK5/6, CDX-2, SATB2, CDH17, GATA3, CEA, B72.3, Ber-EP4, CD10, RCC, CD15, Pax-8, CD117, Dog-1, HMB45, Melan-A, S100, Erg, FLI-1, P63, SMA, TTF-1, GPC3, or Hepatocyte.

### 2.6 Special staining

Tissue sections (4-μm thick) were deparaffinized, washed with xylene, rehydrated in a descending series of alcohol, and washed with distilled water. The slides were oxidized for 10 minutes with periodic acid (batch #: C191201, Zhuhai Basso Biotechnology Co., Ltd), washed with distilled water; stained for 10 minutes with Schiff (batch #: C191201, Zhuhai Basso Biotechnology Co., Ltd), rinsed with running water; then stained for 3 minutes with hematoxylin and rinsed with running water. The slides were dehydrated, transparentized, and sealed with neutral gum. D-PAS was digested with diastase.

**Figure 6:**
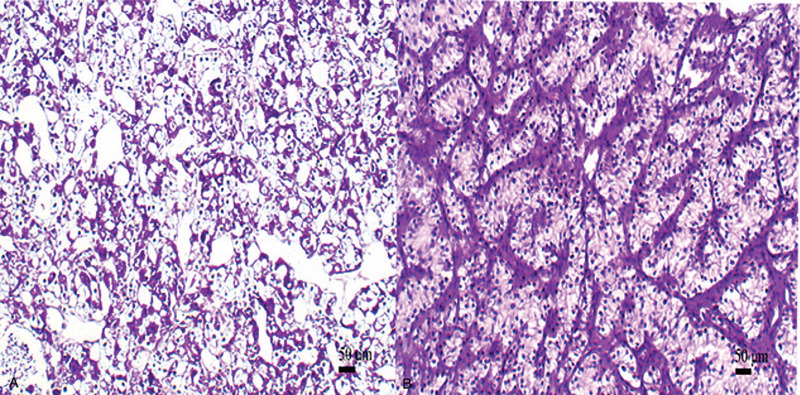
Special staining. (A) Tumor cells were positive for PAS (magnification, ×200). (B) Tumor cells were negative for PAS when digested with diastase (magnification, ×200).

Periodic acid-Schiff (PAS) highlighted intracytoplasmic glycogen deposits (Fig. 6A), which were disappeared when treated with diastase (Fig. 6B).

### 2.7 Whole-exome sequencing (WES) and somatic mutation calling

Tumor and matched normal DNA were extracted using GeneRead DNA FFPE Kit (Qiagen) from formalin-fixed paraffin-embedded (FFPE) tissues. Libraries were constructed by Agilent SureSelect v.4 Kit (Agilent) and sequenced with next-generation sequencing. Genomic DNA was fragmented, end-repaired, adenylated at the 3’ ends, end-connected, amplified, purified, and size-selected in the process of library construction, then sequenced on Illumina X10 platform (Illumina Inc., San Diego, CA).

The WES data were analyzed for mutations and human genome build hg19 was used as reference. Somatic SNVs and In/Dels were analyzed with GATK MuTect2 (version 4.1). The sequenced reads were realigned to hg19 by Burrows-Wheeler Aligner BWA-MEM (http://biobwa.sourceforge.net/) to enhance valid SNVs.

**Figure 7:**
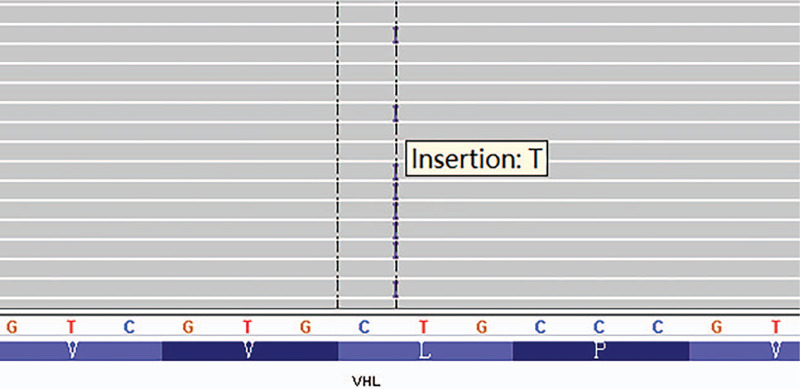
*VHL* mutation site (c.254dupT).

Molecular testing identified a *VHL* gene alteration involving exon 1 (exon 1 c.254dupT, Fig. 7, Table 2).

**Table 2:** Results of gene mutation detection.

Gene	Transcript number	Exon	Base mutation	Amino acid mutation	Mutation abundance	Mutation type
*VHL*	NM_000551	exon1	c.254dupT	p. L85fs	42.90%	Code shift insertion

The reference genome version is GRCh37. Mutation abundance refers to the proportion of the point mutation in total number of wild-type and mutant-type found in the process of gene detection.

**Table 3:** HRD detection result.

Test content	HRD score	Judgment results	Potentially sensitive drugs
HRD	45	Positive	platinum drugs and PARP inhibitor

HRD refers to patients with BRCA harmful mutations or suspected harmful mutations, or patients with genomic instability more than 6 months after the onset of the latest platinum chemotherapy. If there is a BRCA1/2 harmful mutation or suspected harmful mutation and/or a predefined HRD score more than 42, it is defined as HRD positive. If the HRD score is less than 42 and there is no BRCA1/2 harmful or suspected harmful mutation, it is defined as HRD negative. If the HRD analysis fails and the BRCA1/2 analysis is negative, the HRD status is unknown.

Microsatellite instability testing identified microsatellite stabilization (Table 4).

**Table 4:** MSI testing result.

Algorithm	Numerical value	Threshold value	Stability	Result
Step-Wise Difference (DIF)	0.2653	0.4000	Stabilization	Microsatellite stabilization (MSS/MSI-L)
Euclidean Distance (EUC)	0.1439	0.1870	Stabilization	
Cosine Dissimilarity (COS)	0.0319	0.0700	Stabilization	

Microsatellite instability refers to the change of microsatellite length caused by the insertion or deletion of a microsatellite repeat unit caused by DNA mismatch repair defects. It can be divided into MSI stable type (MSS/MSI-L) and MSI highly unstable type (MSI-H).

Tumor mutation burden (TMB) represents the total number of somatic mutations in each MB base of exon coding region in a tumor sample. The calculation formula was as follows: TMB = total number of somatic mutations (including non-synonymous point mutations, insertions and deletions in exon coding region)/target region size, with the unit of mutations/Mb. For this patient, TMB level was low (Table 5).

**Table 5:** TMB testing result.

Test content	TMB numerical value (Number of mutations/Mb)	TMB level	Total percentile
TMB	1.13	low	<80%

Positive correlation index of immune efficacy consisted of *POLE*, *TP53*, *POLD1*, *PBRM1*, *CDKN2A*, *KRAS*, and *DDR* genes. Negative correlation index of immune efficacy consisted of *PTEN*, *EGFR*, *JAK1*, *MDM2*, *JAK2*, *MDM4*, *STK11*, *ALK*, *DNMT3A*, *B2M*, and *CTNNB1* genes. All of these genes were negative.

This experiment detected 18 kinds of neoantigens (Table 6). Twelve frameshift mutations had high affinity (IC50 < 150 nMol). Six were missense mutations. There was no in-frame deletion or in-frame insertion.

**Table 6:** Tumor neoantigens in this patient identified by whole-exome sequencing.

Gene	Mutation type	Mutation amino acid	Mutation location	HLA type	Peptide length	Mutation sequence	Normal peptide affinity	Mutation peptide affinity	VAF
*VHL*	FS	L/LX	85	HLA-B^∗^08:01	9	QLRRRAAAL	NA	4.38	NA
*VHL*	FS	L/LX	85	HLA-B^∗^08:01	10	AQLRRRAAAL	NA	8.62	NA
*VHL*	FS	L/LX	85	HLA-B^∗^58:01	9	AALPNAAAW	NA	8.92	NA
*VHL*	FS	L/LX	85	HLA-B^∗^08:01	9	MAQLRRRAA	NA	11.78	NA
*VHL*	FS	L/LX	85	HLA-B^∗^08:01	10	MAQLRRRAAA	NA	20.22	NA
*VHL*	FS	L/LX	85	HLA-B^∗^08:01	10	QLRRRAAALP	NA	25.83	NA
*VHL*	FS	L/LX	85	HLA-B^∗^08:01	10	RMAQLRRRAA	NA	71.95	NA
*VHL*	FS	L/LX	85	HLA-B^∗^58:01	10	AAALPNAAAW	NA	140.63	NA
*VHL*	FS	L/LX	85	HLA-B^∗^08:01	9	AQLRRRAAA	NA	322.53	NA
*VHL*	FS	L/LX	85	HLA-B^∗^08:01	9	HPQLPRSPL	NA	333.69	NA
*ZNF727*	FS	S/NX	481	HLA-A^∗^32:01	9	NVQRMWQSL	NA	246.72	NA
*ZNF727*	FS	S/NX	481	HLA-B^∗^08:01	9	NVQRMWQSL	NA	439.76	NA
*PLAGL2*	missense	I/L	302	HLA-B^∗^08:01	10	LPMGMYGAHL	426.09	75.79	11.60%
*OTUD6B*	missense	D/Y	70	HLA-B^∗^08:01	10	QLKEKYCALT	157.67	32.34	27.50%
*ARHGEF12*	missense	H/R	393	HLA-A^∗^33:03	9	YLYSDLYKR	1407.05	296.25	61.50%
*OTUD6B*	missense	D/Y	70	HLA-B^∗^08:01	10	DQLKEKYCAL	37.32	10.71	27.50%
*OTUD6B*	missense	D/Y	70	HLA-B^∗^08:01	9	QLKEKYCAL	10.32	4.70	27.50%
*PLAGL2*	missense	I/L	302	HLA-C^∗^07:02	9	MYGAHLPTM	257.38	312.72	11.60%

FS = frame shift, VAF = variant allele frequency.

**Figure 8:**
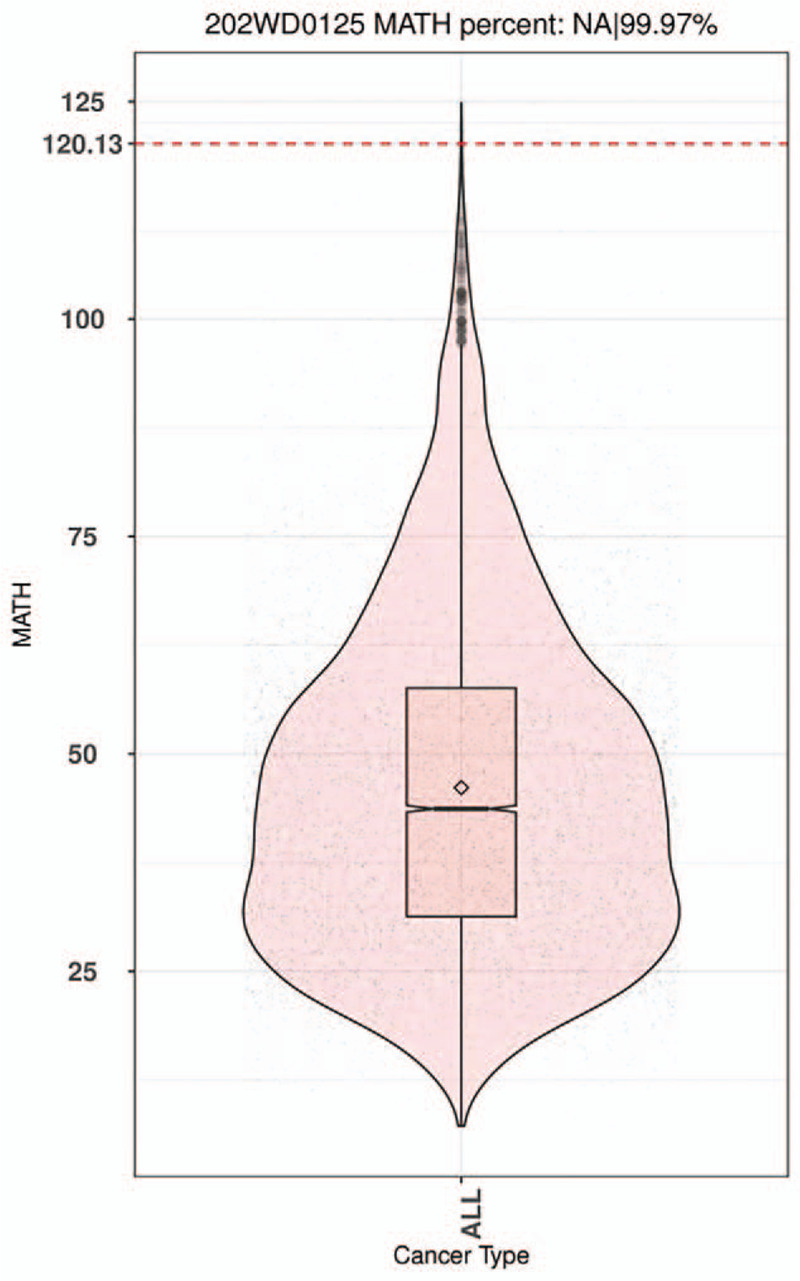
Percentile chart of MATH.

Mutant-allele tumor heterogeneity (MATH) score was used to evaluate heterogeneity. The higher the MATH score indicated the higher the intratumoral heterogeneity (ITH). MATH = 100  × MAD/median.^[6]^ According to the order of examinees in database, MATH values were divided into low (<25%), medium (≥25%, <75%) and high (≥75%). This patient's MATH value was 120.13. Thus, ITH level was high. The Percentile was 99.97% (Fig. 8).

Detection of chemotherapy related genes was as follows. Compared with other genotypes, the subject might be sensitive to methotrexate, cyclophosphamide + epirubicin, fluorouracil or capecitabine based chemotherapy. Toxicity and side effects of cyclophosphamide, cyclophosphamide + doxorubicin, cyclophosphamide + epirubicin, irinotecan, fluorouracil/tegafur + folic acid, fluorouracil + folic acid + oxaliplatin might be relatively low. The subject might be resistant to paclitaxel, cisplatin + paclitaxel, cisplatin + cyclophosphamide, anthracycline, fluorouracil + oxaliplatin and gemcitabine. Toxicity and side effects of platinum compounds + paclitaxel, cisplatin + paclitaxel, cisplatin + cyclophosphamide, anthracycline and pemetrexed may be relatively high. This patient carried no reported genetic pathogenic variation.

### 2.8 Follow up

Before cytoreductive surgery (CRS) + hyperthermic intraperitoneal chemotherapy (HIPEC) operation, the patient received 2 cycles of intraperitoneal (IP) chemotherapy with cisplatin 120 mg and intravenous (IV) chemotherapy with Preto 800 mg. After operation, the patient received IV chemotherapy with docetaxel 60 mg, pemetrexed 750 mg and cisplatin 100 mg, and IP chemotherapy with docetaxel 40 mg. The patient received interventional therapy of hepatic artery embolization for 5 times (Table 7). The patient received no targeted therapy.

**Table 7:** The procedure of treatment.

Date	Event
2018–03	swollen lymph nodes in the right inguinal region
2018–04–11	Laparoscopic exploration and renal peritoneal tumor biopsy
2018–06–18	lP chemotherapy with cisplatin 120 mg and IV chemotherapy with Preto 800 mg
2018–07–13	lP chemotherapy with cisplatin 120 mg and IV chemotherapy with Preto 800 mg
2018–10–18	CRS + HIPEC operation
2018–12–29	IV chemotherapy with docetaxel 60 mg, pemetrexed 750 mg and cisplatin 100 mg, and lP chemotherapy with docetaxel 40 mg
2019–01–25	Chemotherapy with pemetrexed, cisplatin and docetaxel, chemotherapy pump perfusion
2019–02–21	Chemotherapy with pemetrexed, cisplatin and docetaxel, chemotherapy pump perfusion, interventional therapy of hepatic artery embolization
2019–03–20	Chemotherapy with pemetrexed, cisplatin and docetaxel, chemotherapy pump perfusion, interventional therapy of hepatic artery embolization, Take Etan
2019–07–20	Interventional therapy of hepatic artery embolization, liver protection therapy
2019–09–06	Interventional therapy of hepatic artery embolization, liver protection therapy
2019–11–13	Interventional therapy of hepatic artery embolization, liver protection therapy
2020–06–18	The patients increased appetite significantly when he took megestrol acetate dispersible tablets. The reexamination found the gallbladder obstruction. Gallbladder drainage was done. The reexamination found mesenteric nodules increased.
2020–09–04	The patient had the symptoms of incomplete intestinal obstruction. His vomiting aggravated when he took food. He accepted gastrointestinal decompression treatment.
2020–10–05	The patient had the symptoms of incomplete intestinal obstruction. His vomiting aggravated when he took food. He accepted gastrointestinal decompression treatment.
2020–10–14	death

The patient survived over 31+ months and died on October 14, 2020 owing to incomplete intestinal obstruction. This case report was approved by the ethics committee of our hospital. Informed consent was obtained from the patient's son.

## 3 Discussion

Clear cell mesothelioma, also called glycogen-rich or foamy mesothelioma, was considered a variant of epithelioid MM. As an extremely rare entity, it was first described by Ordoñez et al^[7]^ in 1996. It can originate from pleura^[3,4]^ or peritoneum.^[3]^

Clinically, primary peritoneal epithelioid mesothelioma of clear cell type occurs mainly in aged people (Table 8).^[3,8–11]^ The majority of patients were males (male: female ratio of 6:2).

**Table 8:** Literature review of published peritoneal epithelioid mesothelioma of clear cell type.

Author, year	Case	Age/Sex	Follow up, months	(Ref.)
Ordóñez NG, 2005	1	69/M	DOD (4)	^[3]^
	2	61/M	DOD (16)	
	3	67/M	AWD (11)	
Ordóñez NG, 2005	4	67/M	INA	^[8]^
Zannella S, 2014	5	62/M	AWD (10)	^[9]^
Hayashi H, 2017	6	61/F	DOD (2.3)	^[10]^
Smith-Hannah A, 2019	7	63/F	AWD (100)	^[11]^
Current case	8	69/M	DOD (31.6)	

AWD = alive with disease, DOD = died of disease, INA = information not available.

Common characteristics of clear cell peritoneal epithelioid mesothelioma include:

1. abundant clear cytoplasm;
2. evident cytoplasmic membranes;
3. eccentric small round nuclei.

A definitive diagnosis of malignant peritoneal mesothelioma (MPM) requires serial workup, including immunohistochemistry (IHC). Positive IHC markers are Calretinin (tight junction-associated protein), Cytokeratin 5/6 (intermediate-sized basic keratins), WT-1, and D2-40.

To diagnose clear cell peritoneal epithelioid mesothelioma can be challenging owing to a wide variety of tumors with similar morphologic features. Given extreme rarity of such an entity, diagnosis of clear cell mesothelioma could be made after excluding more common intra-abdominal tumors, including carcinomas arising from upper or lower gastrointestinal tract, pancreas, adrenal, kidney, liver and Müllerian system/primary peritoneum, or metastatic carcinomas from breast or lung origins. Other intra-abdominal tumors with clear cell morphology, within the scope of differential diagnosis, included myoepithelial carcinoma, epithelioid leiomyosarcoma, melanoma, perivascular epithelioid cell tumor, paraganglioma, gastrointestinal stromal tumor, epithelioid angiosarcoma, and epithelioid sarcoma.^[11]^ Carcinomas in general were excluded by absent immunostaining for markers such as B72.3, Ber-EP4, and poly-CEA.^[12,13]^

One of the most common genetic alterations in MM was homozygous deletion of 9p21 locus within a cluster of genes spanning cyclin-dependent kinase inhibitor (CDKN)-2A, CDKN2B, and methylthioadenosine phosphorylase. Deletion occurs in approximately 25% of peritoneal MM.^[14]^*BAP1* (BRCA-associated protein 1) mutations or deletions, however, can occur in up to 80% of epithelioid mesothelioma cases.^[15]^ Smith-Hannah et al reported *VHL* gene mutation in clear cell peritoneal epithelioid mesothelioma.^[11]^ We also identified *VHL* gene alteration involving exon 1. The *VHL*, well-known as a tumor suppressor gene, was located in chromosome 3p25. Its protein product, pVHL, performs multiple biological functions.^[16]^

The well-established prognostic factors in MPM were age, histological subtype, completeness of cytoreduction, and disease stage.^[17–20]^ Proliferation measured by Ki67 index has prognostic importance.^[21]^ In our case, the patient's overall survival was 31.6 months, with histology of epithelioid subtype, and Ki67 index of 20%. He underwent cytoreductive surgery and intraperitoneal hyperthermic perfusion chemotherapy.

Most of homologous recombination defects (HRD) were caused by mutations/deletions in homologous recombination repair (HRR) genes. Since genomic damage could not be repaired in time, genomic defects would develop, characterized by chromosome breakage, loss of heterozygosity, and telomere instability. The HRD is evaluated by calculating loss of heterozygosity (hrd-loh), large scale transitions (LST) and number of telomeric allelic imbalances (TAI).^[22]^ This patient had a HRD score of 45, indicating sensitivity to platinum-based chemotherapy and PARP (Poly ADP-ribose Polymerase) inhibitor.

Neoplastic antigens are abnormal proteins, which could be produced by genetic mutations in cancer cells to activate immune system. Neoantigen can be an abnormal polypeptide produced by virus infection, genetic mutation or rearrangement during tumor development and progression. This neoantigen can be presented by major histocompatibility complex (MHC) molecules to activate antitumor immune response. Upon presented by MHC molecule, neoantigen can be recognized, processed and presented by antigen presenting cells (APC) to T cells through specifically binding to T cell receptor (TCR). This process leads to activation, proliferation and differentiation of T cells into cytotoxic effector cells, which initiates anti-tumor immune response.^[23]^ Notably, neoantigens are only expressed on tumor cells with tumor-type specificity. Moreover, sequences of neoantigen peptides are different from normal proteins/peptides, which are not screened by thymus negative selection, so they harbor strong immunogenicity.^[24]^ In addition, heterogeneity of neoantigen is an important factor for survival and prognosis of cancer patients. For example, patients with homogeneous tumors (ITH ≤1%) had a longer overall survival time than those with heterogeneous tumors (ITH > 1%).^[25]^ All of these characteristics indicate that neoantigens could be used as a good target for immunotherapy. Neoantigen-based vaccines have provided a potential complementary therapeutic strategy by increasing immunogenic antigen load, which can enhance tumor-specific immune response. Further research is needed to explore this treatment option in mesothelioma and technological advances are required to translate this concept into clinical practice.^[26]^

In conclusion, primary peritoneal epithelioid mesothelioma of clear cell type is an extremely rare entity. Tumor cells compose of abundant clear cytoplasm, evident cytoplasmic membranes and eccentric small round nuclei. It is necessary to be differentiated from a variety of clear cell tumors. Molecular testing identifies *VHL* gene alteration involving exon 1. Positive HRD indicates sensitivity to platinum-based chemotherapy and PARP inhibitor. Based on WES, 18 potential neoantigens are identified. This patient has a high level of intratumoral heterogeneity. Compared with other genotypes, this subject might be beneficial from methotrexate, cyclophosphamide + epirubicin, fluorouracil or capecitabine based treatment.

## Acknowledgments

The authors thank the patient's son (Chuan-fei Zhu) for providing the treatment process.

## Author contributions

**Formal analysis:** Xue-Mei Du.

**Funding acquisition:** Yan Li.

**Investigation:** Ya-Ping Wei.

**Methodology:** Ying Gao, ZHao Li.

**Resources:** Jian-Mei ZHang.

**Validation:** Hong CHang.

**Writing – original draft:** Xue-Mei Du.

**Writing – review & editing:** Yan Li.
